# Randomized Controlled Study to Test the Effectiveness of Developmental Network Coaching in the Career Advancement of Diverse Early-Stage Investigators (ESIs): Implementation Challenges and Lessons Learned

**DOI:** 10.3390/ijerph182212003

**Published:** 2021-11-16

**Authors:** Mohamed Mubasher, Kimberly Lawson, Priscilla Pemu, Thomas Pearson, Jeffrey Engler, Adriana Baez, Jonathan K. Stiles, Maritza S. Salazar, Lee S. Caplan, Keith Green, Meldra Hall, Muhammed Y. Idris, Ernest Alema-Mensah, Yulia A. Levites Strekalova, Winston E. Thompson, Alexander Quarshie, Elizabeth Ofili

**Affiliations:** 1 Clinical Research Center, Morehouse School of Medicine, Atlanta, GA 30310, USA; klawson@msm.edu (K.L.); pipemu@msm.edu (P.P.); jstiles@msm.edu (J.K.S.); lcaplan@msm.edu (L.S.C.); kegreen@msm.edu (K.G.); meldraphall@gmail.com (M.H.); myidris@msm.edu (M.Y.I.); eamensah@msm.edu (E.A.-M.); wthompson@msm.edu (W.E.T.); aquarshie@msm.edu (A.Q.); eofili@msm.edu (E.O.); 2 College of Public Health and Health Professions, University of Florida Gainesville, Gainesville, FL 32611, USA; tapearson@ufl.edu (T.P.); yulias@ufl.edu (Y.A.L.S.); 3 Council of Graduate Schools, Washington, DC 20036, USA; jengler@cgs.nche.edu; 4 Medical Sciences Campus, University of Puerto Rico, San Juan 00921, Puerto Rico; adriana.baez@upr.edu; 5 Paul Merage School of Business, University of California, Irvine, CA 92697, USA; maritza.salazar@uci.edu

**Keywords:** diversity, early-stage investigators, career development, nested cluster randomization, mentorship, developmental networks, National Research Mentoring Network (NRMN), COVID-19, grant writing, mock study section

## Abstract

**Introduction:** Adding developmental networks (DN) to grant-writing coaching can significantly enhance ESIs’ research careers. Herein, we present study design, ESIs’ characteristics and encountered challenges/lessons learned and their resolutions when deploying/implementing (a) NCR algorithm(s), (b) recruitment/retention and (c) implementing DN intervention. **Methods:** Nested Cluster Randomization (NCR) design governs this study implementation. The sample size is 220 ESIs intending to submit an NIH K, R, U, and/or Minority Supplement application(s). Primary outcome: intensity/sustainability of grant submission(s)/funding(s), measured by time to/between application(s). Outcome(s) analyses modes: summaries, Kaplan Meir and Cox proportional hazard models as a function of randomization groups and other predictors of outcomes. **Results:** In the present study, we recruited two cohorts of ESIs (N = 85): 39% African Americans, 18% Latinx, 18% Whites, 20% Asians and 6% Hawaiian/Pacific Islander/other ethnicities; 65% are women; 73% are assistant professors, 4% are Associate Professors and 23% are instructors/scientists/post-doctoral. Participants’ disciplines: 32% basic/biomedical, 36% clinical/translational and 32% social/behavioral. Proposal(s) mechanisms: 61% research grants (R series), 31% career development (K series), 7% support of competitive research (SCORE) and 1% National Science Foundation applications. NCR did produce balance in the distribution of ESIs’ demographics, sex at birth, ethnicity, professional appointments, background disciplines, and mechanism of sought funding. **Lessons learned/challenges:** NCR implementation was methodologically challenged during implementation by added constraints (e.g., assigning coaches to the same randomization arm of their participants as well as blinding them to ESIs’ randomization group). Recruitment and retention were hampered by the COVID-19 pandemic and more progressive and innovative strategies were needed to heighten the visibility and outreach of this program. DN delivery was also affected by the pandemic and monitoring of ESIs’ engagement and facilitation of communications interventions were needed. Resolution of these challenges effectively reconfigured NCR algorithms, recruitment/retention plans, and DN intervention delivery. We intend to recruit an additional 135 ESIs focusing on underrepresented scholars from RCMIs, CTSAs, and other programs. COVID-19 rendered this program 100% virtual, with recruitment/retention challenges and substantial disruption of ESIs’ research. We may extend the grant writing period, coaching, and Mock Study Section support.

## 1. Introduction and Background

The development of the research skills of Early-Stage Investigators (ESIs) has been at center stage of many academic institutions in the USA [1,2,3]. Mentoring and training to develop the research skills of ESIs are important strategies for facilitating faculty success [4]. However, research studies suggest that scientists from underrepresented groups are not equally engaged in mentoring relationships and often have less access to quality traditional dyadic relationships [5]. Ginther et al. also observed a disturbing discrepancy in success rates for research grant (R01) applications between White and Black applicants, even after adjusting for numerous observable variables [6]. In a recent integrative literature review by Ransdell et al., 2021 [4] that was based on 46 PsychINFO, CINAHL, and PubMed published papers in English between 2010 and 2020, the authors reported barriers to research development among ESIs from underrepresented minority faculty that included bias, discrimination and isolation as well as an institutional lack of mentors and devaluation of experience or expertise.

Developmental Networks (DN) can potentially play a critical role in the career progression of under-represented early scientists by affecting their science identity and improving their self-efficacy [7]. The Diversity Program Consortium (DPC) supports the implementation and assessment of mentoring and training interventions that could improve recruitment, retention and advancement of investigators’ careers from diverse backgrounds, including those from underrepresented groups in the basic biomedical, behavioral, clinical, and social sciences [8,9].

In order to develop and sustain quality mentorship of diverse ESIs, we launched the study as the NIH-sponsored National Research Mentoring Network (NRMN) Strategic Empowerment Tailored for Health Equity Investigators (SETH), which seeks to evaluate the impact of an intervention that addresses the developmental networks of diverse ESIs. According to the NIH, an ESI is an investigator on a research track to becoming a Program Director or a Principal Investigator (PD/PI) after completing his/her terminal research degree or end of post-graduate clinical training (whichever date is later) within the past 10 years and who has not previously successfully competed as a PD/PI for a substantial NIH independent research award (Investigator Career Stage Benefits|NIH Center for Scientific Review). For the purpose of this study, ESIs are investigators on a research career track who have not yet received independent NIH R01 or equivalent funding.

The primary underlying research hypothesis of this study is that DN significantly accelerates the research track of ESIs towards becoming independent investigators, i.e., PD/PI on an NIH R01 or equivalent funded grant.

To set the analysis stage for testing the hypothesis of this study, we deployed a cluster randomization design [10,11,12,13,14,15,16] to recruit and randomly assign ESIs to either the control (structured grant writing coaching alone) or the intervention group (structured grant writing coaching plus mentoring to developmental network of ESIs).

**Figure 1:**
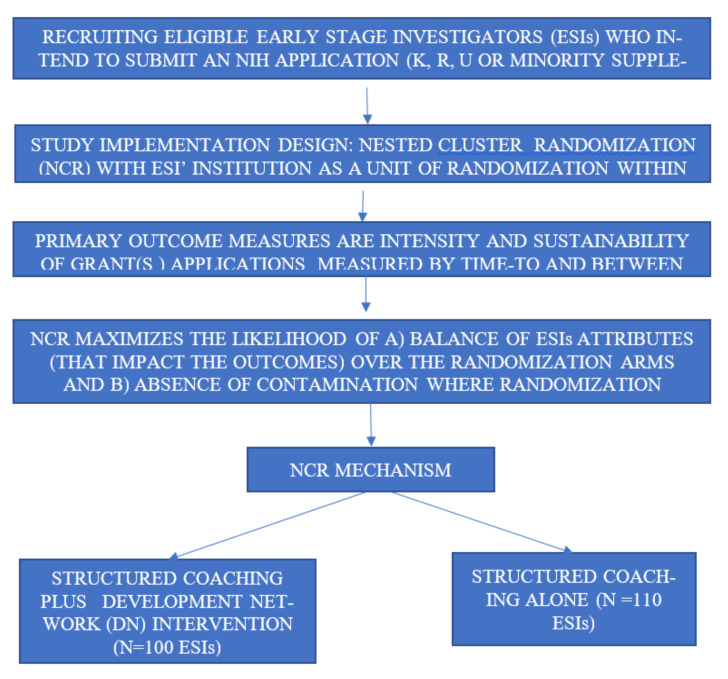
Testing the efficacy of development network (DN) among ESIs in a structured grant writing coaching training.

The ESIs’ role in the study is to work (within their institutions) closely with their study- assigned coaches and, if randomized to the DN group, the network study-assigned developer, to develop and submit application(s) for funding from NIH (K, R, U and/or minority supplements/NSF, within 12 months of recruitment into the study (Figure 1).

Historically, cluster randomization designs are known to be scientifically and methodologically suited for feasible administration (of randomization assignments) and to minimize bias resulting from randomized groups interacting with each other (contamination) [11,12,13,14]. Additionally, they invoke a randomization mechanism that targets the goal of balancing the underlying variables that are assumed to be correlated with the primary outcome measure of the research study [10,11,12,13,14,15,16]. Lack of balance of the distribution of these variables across the study arms/groups can potentially introduce bias in the study results.

The study was designed to evaluate the effectiveness of the intervention to improve the developmental networks (DN) plus structured grant writing coaching (intervention), relative to structured grant writing coaching alone (control), on the research productivity of ESIs (Figure 1). The study also aims to test the independence assumption of the institutional setting from the ESI’s ability to train and access developmental networks to advance their research career.

The work in this manuscript describes the underlying study design and rationale. It also characterizes the demographic and professional distributions of the study cohorts’ ESI scholars (thus far recruited) in terms of sex at birth, ethnicity, background disciplines, academic professional titles, and the mechanism of anticipated funding. Additionally, in implementing this study, we listed the challenges encountered and lessons learned in deploying the cluster randomization scheme, recruitment and retention and delivery of the DN intervention.

## 2. Methods

A nested cluster randomization algorithm guided the experimental design underlying the implementation of this study.

***Cluster Randomized Clinical Trials (CRCT) Designs***

### 2.1. Overview

Typically, clinical trials use individual participants as a unit of randomization to investigate significant differences in outcomes between different study arms. However, at times, individual allocations are not possible or desirable; therefore, groups/clusters of individuals are randomized instead [10,11,12,13,14,15,16]. Lindquist [10] initially proposed the concept and methodology of cluster (group) randomization in 1940. In this methodology, cluster trial groups of participants (e.g., in different clinics, institutions, neighborhoods, etc.) are the unit of randomization. Cluster trials are increasing in popularity among health service researchers [12,13]. One of the methodologically appealing features of CRCT is that participants within clusters are usually homogeneous with respect to the factors that may affect or correlate with the outcome(s) of the study. This feature makes it easier to decipher effects (measured by the outcome) if they do exist. One of the underlying reasons for deploying CRCT is feasibility. An example of this is vaccine trials to prevent a communicable disease, which require cluster randomization to minimize the ‘herd’ effect [14]. Another reason for employing CRCT designs is to avoid “contamination” between those receiving the intervention and those who are not. Contamination potentially “dilutes” the effects of the intervention (dilution bias), resulting in an increase in Type II errors (false negative error). While CRCTs are generally robust, they require larger sample sizes than RCTs, with the individual as the unit of randomization. The inflation in sample size is usually caused by incorporating an estimate for the intra-class correlation among within-cluster individuals [15,16].

### 2.2. Study Experimental Design, Implementation of the Nested Cluster Randomization and Analyses Plans

The study implementation approach is based on a blinded (whenever it is logistically feasible for coaches and the project investigators) nested cluster randomization design. The unit of the nested cluster randomization is the ESI scholar’s institution. This design will test the earlier-stated study hypothesis by increasing the likelihood of isolating the effects (or lack of it) of the intervention vs. the control. This potential is primarily achieved through the random nature of assigning institutions (within which the ESI scholars are nested) to either study arm. Additionally, the design also ensures equal distribution of measurable and non-measurable factors underlying the participant’s ability to attain professional growth, such as prior years of professional experience, self-efficacy and motivation, the existence and quality of engagement with mentors, etc. Furthermore, our adopted nested cluster randomization enhances the likelihood of minimizing the potential for contamination that may result from members of the different study arms (particularly the DN plus structured coaching group) interacting and sharing specifics of their training and knowledge with members of the control group.

The study’s primary outcome measure/metric is the intensity and sustainability of grant submission(s) measured by time to and between submission(s) of the grant application(s) to NIH funding mechanisms (K, R, U, and minority supplements). The secondary outcomes include the number of grant awards, number of scored grants, number of publications in peer-reviewed journals, ESI developer network measures including size, composition, and the structure of each ego network, plus self-efficacy and career progression in academic or other non-academic research.

The study’s statistical analysis plan and design ultimately targets testing the null hypothesis of equal effects between the randomized study groups in the primary and secondary outcome measures, versus the alternative hypothesis that one group is significantly superior to the other. A total sample size estimate of 220 participants was determined to afford the study 90% statistical certainty/power to detect differences between the control and the intervention groups, if they truly exist. Additional considerations included a stipulation of a 0.05 alpha/significance level (the predetermined threshold of falsely rejecting the null hypothesis), an assumed 0.2–0.3 effect size for the intervention vs. the control group, and a 20% attrition/loss to follow up rate. Analyses of the study outcome measures will follow the intent-to-treat principal.

The statistical methods for this report focus on testing the balance of participants’ attributes between the intervention and the control arms. Frequencies and percentages summarized categorical attributes. Univariate tests of the balance of baseline participants’ categorical characteristics between randomization groups were based on the Chi-square/Fisher exact tests. Analyses and graphics used SAS version 9.4, and R.4.0.0. Overall significance level was preset at 0.05.

***Study coaches and network developers:*** For cohorts 1 and 2, the study recruited 12 coaches and 6 network developers. Experienced investigators were recruited as coaches to match the scholars’ background disciplines (basic/preclinical science, clinical/translational research, and behavioral and social sciences.) It is worth noting that the study’s coaches are blinded to their scholars’ study arm randomization assignment. Furthermore, to avoid contamination, we assigned coaches to the same randomization arm of their participants. Network developers were not recruited to match the participants’ background, but rather to provide personalized feedback to the ESIs about how to improve the quality of their research network and mentorship ties regardless of their field of study.

***Regular Mock NIH-like Study sections:*** This study implements full study section reviews to advise ESIs with completed applications. The sessions are spearheaded and orchestrated by a senior study investigator, a subject-matter expert reviewer, and a biostatistician.

## 3. Results

### 3.1. Participant’s Academic and Demographics Characteristics

Table 1 shows the distribution of participants’ characteristics by randomization groups (denoted as “1” and “2”). Thus far, two cohorts totaling 85 ESI participants have been recruited, corresponding to 39% of the targeted total sample size. They represent diverse racial/ethnic groups comprising 39% Black/African American, 18% Latinx, 18% Whites, 20% Asians, and 6% Hawaiian Pacific Islander or other ethnic origins. The majority of the ESI participants (65%) are females. Sixty-two (73%) are assistant professors, three (4%) are associate professors, seven (8%) instructors/scientists, and thirteen (15%) post-doctoral trainees. Their background disciplines comprise 27 (32%) basic biomedical sciences, 31 (37%) clinical or translational, and 25 (29%) social or behavioral sciences. The target mechanism of funding by the ESI participants are 61% R series awards, 31% K series awards, 7% SCORE awards, and 1% NSF applications.

### 3.2. Balance of Participants’ Academic and Demographics Characteristics between the Control and the Intervention Groups

**Figure 2:**
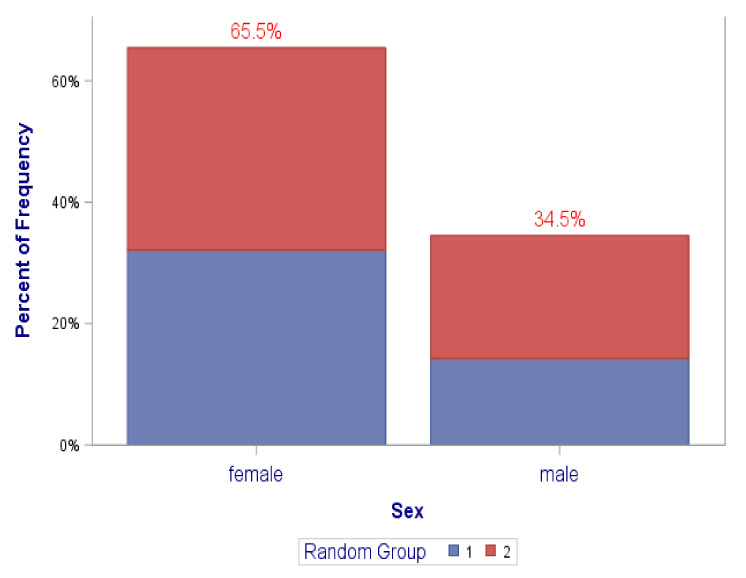
Distribution of gender by randomization groups (1,2) (*p*-value = 0.3956).

**Figure 3:**
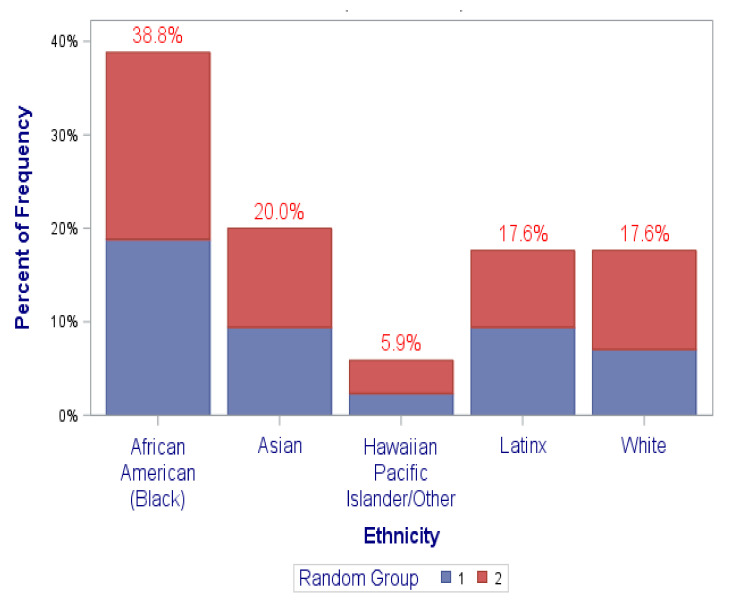
Distribution of ethnicity by randomization groups (1,2). (*p*-value = 0.9227).

**Figure 4:**
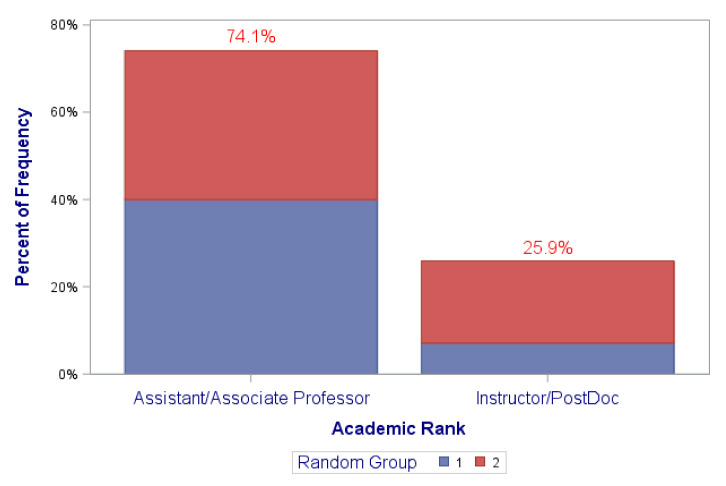
Distribution of academic position by randomization groups (1,2). (*p*-value = 0.3009).

**Figure 5:**
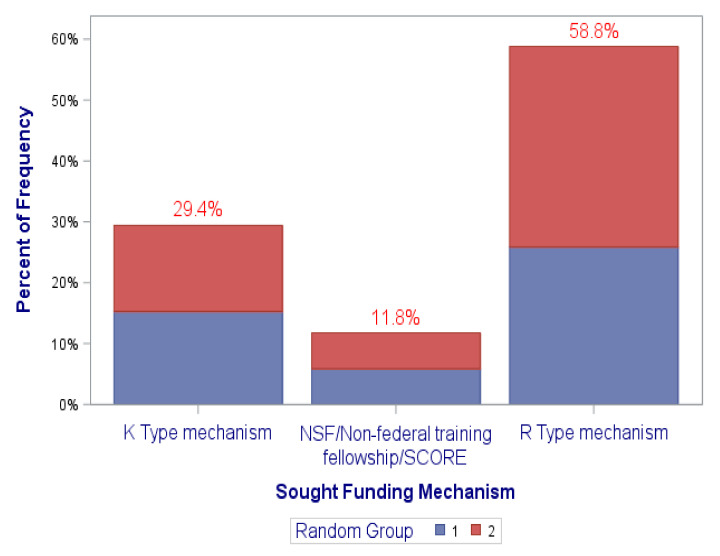
Distribution of sought funding mechanism by randomization groups (1,2). (*p*-value = 0.1575).

Figure 2, Figure 3, Figure 4 and Figure 5 graphically illustrate the balance between the “coaching alone” and the coaching plus “DN” randomization groups in the distributions of the frequencies of the covariates of (1) sex at birth, (2) race/ethnicity, (3) professional academic positions, (4) ESIs participants’ background disciplines, and (5) sought mechanism of funding. This is displayed by the semi-equivalence of the two colored areas in the graph’ bars for each level/subgroup of each covariate (except perhaps for sex at birth and sought mechanism of funding, though they still did not reach statistical significance). As such, each color represents the total frequency of those in either randomization groups, denoted by group “1” or “2”. It is worth noting, however, that there were no statistically significant differences between the study randomization groups in these covariates. All *p*-values were > 0.05 (Table 1 and Figure 2, Figure 3, Figure 4 and Figure 5).

## 4. Challenges and Lessons Learned Discussion

### 4.1. The Implementation of the Pursued Nested Cluster Randomization Design

As mentioned earlier, we implemented institution-based cluster randomization, with the study respective ESIs nested within the institutions. We purposely appealed to this randomization method to balance the distribution of ESIs across the (randomization) groups with respect to the outcome predictors and simultaneously minimize the potentials of contamination/randomization groups’ interactions. In what follows, we will describe the challenges we faced in developing and implementing the randomization algorithm(s) and how we remedied and met these challenges.

***Statement of the Problem and challenges:*** We have two randomization arms, denoted by *Arm_s_*, s = 1, 2. The unit of nested cluster randomization is the ESI’s institution**s,** which we denote by Inst*_i_, i* = 1, 2,…, *n.* The study ESIs are layered within the institution so that Inst*_i_(*ESI*_j_) j* = 1, 2,…, *n_i_* now becomes the ultimate unit of randomization. ESIs are recruited in batches (cohorts), i.e., different groups are sequentially recruited at different time points. By design, we blinded the “Control” coaches (who are a separate group from the Intervention coaches) to the randomization assignment of the ESIs. Notice that, by virtue of selecting the institution as the unit of randomization and to minimize contamination, that institution automatically becomes *locked* to the initial randomization assignment as long as there is (within that institution) an inter-cohort overlap of ESIs (i.e., at least two scholars from different cohorts at the same institution). This feature, coupled with the fact that recruitment of ESIs is staggered by cohort (different cohorts are recruited at sequential but different time points), consequently, renders recruitment of ESIs to become non-uniform (i.e., not of equal sample size) within the different institutions, which potentially creates non-uniformity with regard to the number of ESIs per randomization group. It is also worth mentioning that for the study’s initial (and subsequent) cohort(s), we closely matched, by background discipline (clinical/translational, basic/genetic, and social/behavioral), the coaches to the ESIs. We also further preserved, within coaches, the selection of the ESIs to be of the SAME randomization arm. In other words, for each coach, we assigned ESIs who were randomized to the same group. Now, for subsequent cohorts, and as long as there is an inter-cohort overlap of ESIs within the coach, that coach is consequently *locked* to the randomization arm assignment of the ESIs in his/her group. This now presents an added constraint when assigning coaches to additional ESIs.

***Remedies***: These challenges and constraints were resolved by adapting (with modifications) the algorithm for randomization using the “CVCRAND” package [17]. This (R.4.0 software) package was developed to perform constrained randomization on the clusters (e.g., Institutions) into two arms with an option to use user-defined weights on the covariates. Some of the institution-level covariates we used with this package was whether the institution is among the top 50 NIH-funded for 2019–2020 (Yes/No) [18] and Carnegie Classifications [19]. To ensure balance between the two randomization groups, we also used the balance score for constrained randomization [20]. Our ultimate goal was to balance the distribution of the study outcomes’ predictors between the two randomization groups, including preservation of the following equality:

Probability of Inst*_i_(*ESI *_j_) belonging to Arm_s_*to be EQUAL to 0.5, which is also equals **to**

Probability Inst*_l_(*ESI *_m_) belonging to Arm_t_*where *i, j* and *s* are separately not equal to *l, m* and *t*.

To check the randomization validity [21], we used the argument of “check_validity” in both cvrall() and cvrcov() functions, which was set to be TRUE. This would produce summary statistics on cluster (institution) pairs that always or never appear together in the same arm, which ascertain the validity of randomization.

### 4.2. ESIs Recruitment and Retention

The target audience for recruitment were postdoc, junior faculty, specifically assistant professors and instructors; clinicians at the associate professor level who are new to research are also eligible. Applicants were recruited through multiple networks: Research Centers at Minority Institutions (RCMI), Clinical and Translational Science Award (CTSA) institutions, the National Research Mentoring Network, and the Association for Academic Minority Physicians (AAMP). The announcement for the research study was created by the NRMN-SETH staff and distributed as an email flyer to the multiple networks. Application links, study participation eligibility, study benefits, and study timeline were all included in the NRMN-SETH flyer. In addition, RCMI Principal Investigators at the 21 awardee institutions were emailed and asked to nominate junior faculty members from their respective institutions to participate in the research study.

Retention of the 60 study participants from cohort 1 was very challenging as 13 study participants withdrew during the first three months of the research study. At the onset of the COVID-19 pandemic, 10 more participants withdrew as campuses began to shut down in the Spring 2020, forcing many of our study participants to teach remotely and abandon/postpone their research projects at the campus laboratories. The increased faculty workload along with responsibilities for balancing work, home, and family were often cited by participants as primary causes for their withdrawal from the research study. The NRMN-SETH team administered a survey in August 2020 to assess the impact of COVID-19 on our cohort 1 study participants. The specific areas of impact included meeting grant submission deadlines, sustaining communication with SETH coaches and coaching groups, stress, career transition, self-efficacy and management of scholarly tasks, overall confidence to meet career challenges, and effects of their family situation on professional progress (manuscript under review).

To better accommodate cohort 1 study participants, we have allowed these scholars to return in subsequent cohorts to complete the coaching group experience and allowed others submitting their grant application at a later time than anticipated to participate in a mock study section prior to submission although the cohort has ended.

To help with our future recruitment efforts, we plan to maximize and collaborate with the W. Montague Cobb/NMA Health Institute, which is headquartered in Washington D.C. They focus on identifying issues and developing solutions that will reduce racial and ethnic health and healthcare disparities and improve the health of all Americans. They will assist us with the distribution of our study fliers and identify if their participants are a good match for our program, and this will greatly increase our recruitment efforts. In addition, we plan to host webinars with them to showcase what our program has to offer.

Leveraging social media is another way we will disseminate our information. This is conducted is with the aim of attracting potential participants to contact our team for more information and for consideration of enrollment.

In recruiting cohort IV for this study, two strategies were implemented to address and mitigate the recruitment challenges experienced with earlier cohorts. In interviewing participants, it became clear that push-communication strategies through targeted emails and outreach are more effective than pull-communication through the NRMN website and broad program opportunity announcements (Smith, 2018). Therefore, we have queried the NIH Reporter for the PI contact information of (1) PIs of active research grants with supplements and (2) PIs of career development grants (e.g., K01, K08, K99/R00). The former represents a group of investigators committed to mentoring and career development of junior investigators, and the former are the junior faculty who are the primary audience for the NRMN-SETH program. We harvested the public email addresses of PIs and sent personalized emails inviting them to either share the information about the program with their mentees or submit an application as a participant. To provide further opportunities for engagement with the program, we scheduled several informational seminars. The seminars were well-attended and allowed prospective coaches and participants to learn more about the intent of the program and to ask questions. Similar to the email outreach strategy, we encouraged webinar participants to share the information within their professional contacts and allow us to reach out to the second- and third-degree networks. At the time of the writing of this paper, we have received three times the number of applications compared to the number of participant slots available in the program.

### 4.3. Implementation of the Developmental Network Coaching

Scholars in the experimental arm of the developmental network randomized control study participate in five one-hour-long webinars. During these webinars, important topics related to developing and fostering their relationship with individual mentors forming the mentoring committee, such as effective networking and mentoring up, were discussed. Additionally, scholars also had at least three one-on-one meetings with developers about their specific goals to strengthen their professional developmental network to become a successful and competitive researcher. These above-mentioned activities, along with developmental goals and aspirations of the scholar, are all tracked and monitored over time via a shared repository. The growth and expansion of the developmental network of each scholar is assessed with pre and post intervention surveys. One challenge to assessing the effectiveness of the growth of developmental networks of scholars in the experimental conditions compared to the control group was that it is unclear whether individuals in the comparison group might have received professional developmental opportunities that covered similar learning topics. To mitigate this effect, we will be asking all individuals to share their other professional growth endeavors and activities.

### 4.4. NIH-Like Mock Reviews to Enhance Scientific Rigor of ESIs Applications for Funding

As mentioned in the methods section**,** regular mock NIH-like study sections were implemented to advise ESIs with completed applications on all aspects of their proposals including feasibility, design and hypothesis, significance, innovation, conceptual framework, methods, and research strategies. The reviewing committee comprises a senior study investigator, a subject-matter expert reviewer, and a biostatistician. Initially, and for the first study cohort, ESIs were advised to submit (on a voluntary basis) their applications to the mock reviews prior to submission to funding agencies. By the end of recruitment of the first study cohort, our data indicated that 20% of those who elected to go through a mock review vs. 16.7% who did not were awarded funding. The corresponding, respective percentages for those who applied for funding was more disparate (60% vs. 16.7%).

These statistics prompted the leadership of the study to mandate mock review sessions prior to submission of applications for all scholars recruited after the first cohort.

## 5. Discussion, Conclusions and Future Directions

Rigorous methodology on the evidence base for implementing developmental networks is relevant to the NIH focus on diversity, equity, and inclusion. This paper describes challenges to implementing a randomized controlled study design. The paper describes how we addressed these challenges while maintaining the rigor of the study design. Our findings are important for the ongoing implementation of current NRMN-SETH studies and should inform future studies on the career advancement of diverse scholars.

### 5.1. Balance of ESIs Characteristics between Randomization Groups

The nested cluster randomization scheme using the institution (within which participants are nested) as a unit of randomization, did produce non statistically significant differences indicating balance of the ESI characteristics distributions between the study randomization groups (DN plus coaching vs. coaching alone).

### 5.2. Preservation of Blinding and Minimization of Contamination (Interaction of ESIs across Randomization Groups)

Based on regular meetings between the study senior personnel and coaches, no evidence of unblinding was detected. Additionally, according to regular study network developers’ reports, no evidence of contamination was established, i.e., it was not determined through regular encounters between the developers and their assigned ESI participants that a professional interaction occurred with ESI scholars from the coaching alone group.

### 5.3. Magnitude of Differences in Frequencies between Randomization Groups

Though no statistically significant differences were detected in the baseline characteristics of the scholars between those randomized to the control and the intervention groups, some notable distributional numerical differences occurred (e.g., number of females, African Americans, and “k” type of sought mechanism of funding). This is mainly attributed to the interim nature of this report, where only about one third of the ESI participants have been recruited according to the cluster randomization design. We expect at the end of recruitment closer numerical and frequency distributional results between the two randomization groups.

### 5.4. Retention Challenges and the Impact of the COVID-19 Pandemic

The pandemic posed a significant challenge for scholars to engage with their coaches and DN mentors, which resulted in considerable delays in pursuing completion of applications. Twenty-three (27%) scholars withdrew from the study (control: n = 11, intervention: n = 12). Twelve scholars withdrew after the occurrence of the COVID-19 pandemic.

### 5.5. Future Plans and Implications

**Recruitment vis à vis COVID-19 Challenges**

We intend to recruit an additional 135 ESIs focusing on underrepresented scholars by targeting Research Centers at Minority Institutions (RCMI), CTSAs, and other national research programs. Due to COVID-19, this program is now 100% virtual. The pandemic also posed challenges to recruitment and retention and caused interruptions and delays for the project’s ESI scholars in their research projects and grant writing. Therefore, we are considering extending the timeframe for grant writing/coaching and mock study sections for the ESI scholars, with attention paid to preserving the integrity of the randomization and to avoiding contamination between the groups.

**Nested Cluster Randomization Scheme**

***Lessons-learned informed practical ramifications***

We will closely monitor the Nested Cluster Randomization scheme and test its fidelity on an ongoing basis in terms of balancing characteristics of the ESIs between randomization groups and minimization of contamination.

***Lessons-learned informed theoretical ramifications***

We started on a simulation-based project utilizing R-4.0 and Statistical Analysis System (SAS) version 9.4 Statistical Software to provide an optimal algorithm to guide investigators and statisticians on future implementations of NCR. The statistical computation project is targeting resolution(s) of the challenges encountered in our implementation of the NCR scheme, namely the several constraints imposed by (1) the necessity of minimizing contamination (one randomization group interacting with another), (2) *blinding* of the study coaches to the randomization group of their assigned ESIs, (3) assigning coaches to the same randomization arm of their ESIs’ participants, and (4) balancing the number of assigned ESIs and their respective predictors of study outcomes between the two study groups. Our intent is to publish such work, once completed, as a practical guideline on NCR implementation.

***Lessons-learned informed practical ramifications on recruitment strategies***

To enhance our recruitments efforts, we intend to target outreach programs and as such, we will capitalize on second- and third-degree connections among professional networks. An additional strategy that we also targeted was to leverage social media to heighten the visibility of our SETH program. Such strategies have already yielded fruits by tripling the number of applicants to this program.

***Developmental Network***

We implemented protocols that closely monitor ESIs’ engagement with their assigned network developers to efficiently intervene in resolving communication issues and other ESIs-specific challenges that may potentially diminish their interactions. Such *timely* focused interventions can support the professional development of the scholars by maximizing their utilization of the developmental networking available resources. Furthermore, ongoing monitoring of the short and long-term effects of “cultivating developmental networks” will provide more evidence of the benefits of our training and development efforts.

## Figures and Tables

**Table 1:** Baseline Characteristics and Attributes of Study Recruited ESI Scholars by Randomization Groups.

	All				
	Random Group				
	1		2		
	N	%	N	%	*p*-Value
All	40	100.00	45	100.00	
**Sex at Birth**					0.3966
Female	23	57.50	32	71.11	
Male	16	40.00	13	28.89	
Not Reported	1	2.50	-	-	
**Ethnicity/Race**					0.9227
African American (Black)	13	32.50	20	44.44	
Asian	7	17.50	10	22.22	
Hawaiian Pacific Islander/Other	2	5.00	3	6.67	
Latinx	8	20.00	7	15.56	
White	10	25.00	5	11.11	
**ESIs’ Application Disciplines**					0.1575
Basic Science	12	30.00	15	33.33	
Clinical and/or Translational Science	14	35.00	17	37.78	
Social/Behavioral Science	14	35.00	13	28.88	
**Professional Titles**					0.4740
Assistant	31	77.50	31	68.89	
Associate	2	5.0	1	2.22	
Instructor/Post Doc/Other	7	17.50	13	28.89	
**How much postdoctoral research training have you had?**					
< 1 year or none (n = 1 per group)	30	75.00	32	71.10	
1–3 years	6	15.00	10	22.23	
More than 3 years	4	10.00	3	6.67	
**Sought Funding Mechanism**					0.1022
K Type mechanism	8	20.00	17	37.78	
NSF/Non-federal training fellowship/SCORE	7	17.50	3	6.67	
R Type mechanism	25	62.50	25	55.56	
**What is the highest degree you have obtained?**					0.8464
MD-PhD	3	7.50	1	2.22	
PhD	34	85.00	42	93.33	
Other	3	7.50	2	4.44	

## References

[1] School of Medicine and Dentistry, University of Rochester. Faculty Development, Education, University of Rochester Medical Center, Rochester, New York. The Rochester Early-Stage Investigator (RESIN) (Rochester Early-Stage Investigator Network Program (RESIN).

[2] Penn State Clinical and Translational Science Institute Early-Stage Investigator Training Program (KL2). psu.edu.

[3] University of Minnesota, Neurology Research Development Early Stage Investigator|Neurology Research Development. umn.edu.

[4] Ransdell L.B., Lane T.S., Schwartz A.L., Wayment H.A., Baldwin J.A. (2021). Mentoring New and Early-Stage Investigators and Underrepresented Minority Faculty for Research Success in Health-Related Fields: An Integrative Literature Review (2010–2020). Int. J. Environ. Res. Public Health.

[5] Urizar G.G., Henriques L., Chun C.-A., Buonora P., Vu K.-P.L., Galvez G., Kingsford L. (2017). Advancing research opportunities and promoting pathways in graduate education: A systemic approach to BUILD training at California State University, Long Beach (CSULB). BMC Proc..

[6] Ginther D.K., Schaffer W.T., Schnell J., Masimore B., Liu F., Haak L.L., Kington R. (2011). Race, ethnicity, and NIH research awards. Science.

[7] Yip J., Kram K.E. (2017). Developmental Networks: Enhancing the Science and Practice of Mentoring. http://phd.meghan-smith.com/wp-content/uploads/2016/01/3-Developmental-Networks.pdf.

[8] McCreath H.E., Norris K.C., Calderόn N.E., Purnell D.L., MacCalla N.M.G., Seeman T.E. (2017). Evaluating efforts to diversify the biomedical workforce: The role and function of the Coordination and Evaluation Center of the Diversity Program Consortium. BMC Proc..

[9] Gibbs K.D., McGready J., Griffin K. (2015). Career Development Among American Biomedical Postdocs. CBE Life Sci. Educ..

[10] Lindquist E.F. (1940). Statistical Analysis in Educational Research.

[11] Cook A.J., Delong E., Murray D., Vollmer W.M., Heagerty P.J. (2016). Statistical Lessons Learned for Designing Cluster Randomized Pragmatic Clinical Trials from the NIH Health Care Systems Collaboratory Biostatistics and Design Core. Clin. Trials.

[12] Donner A., Klar N. (2000). Design and Analysis of Cluster Randomization Trials in Health Research.

[13] Bland J. (2004). Cluster randomized trials in the medical literature: Two bibliometric surveys. BMC Med. Res. Methodol..

[14] Puffer S., Torgerson D.J., Watson J. (2005). Cluster Randomized Controlled Trials. J. Eval. Clin. Pract..

[15] Shulzm K.F. (1995). Subverting randomization in controlled trials. J. Am. Med. Assoc..

[16] Kjaergaard L.L., Villumsen J., Cloud C. (2001). Reported methodologic quality and discrepancies between large and small randomized trials in meta-analyses. Ann. Intern. Med..

[17] Yu H., Li F., Gallis J.A., Turner E.L. (2020). Cvcrand Package R 3.6.2. https://cran.r-project.org/web/packages/cvcrand/cvcrand.pdf.

[18] NIH Awards by Location & Organization Top 50 NIH-Funded Institutions of 2020. https://report.nih.gov/award/index.cfm?ot&fy=2010&state&ic&fm&orgid&view=stateorg&sumcol=fun&sumdir=desc.

[19] About Carnegie Classification. The Carnegie Classification of Institutions of Higher Education; Indiana University Center for Postsecondary Research. Retrieved 2 June 2017. https://academicinfluence.com/resources/guidance/carnegie-classifications-college-tiers.

[20] Raab G.M., Butcher I. (2001). Balance in cluster randomized trials, Statistics in Medicine. Stat. Med..

[21] Bailey R.A., Rowley C.A. (1987). Valid randomization. Proc. R. Soc. Lond. Ser. A Math. Phys. Sci..

